# Retreatment with HBV siRNA Results in Additional Reduction in HBV Antigenemia and Immune Stimulation in the AAV-HBV Mouse Model

**DOI:** 10.3390/v16030347

**Published:** 2024-02-23

**Authors:** Ellen Van Gulck, Nádia Conceição-Neto, Liese Aerts, Wim Pierson, Lore Verschueren, Mara Vleeschouwer, Vinod Krishna, Isabel Nájera, Frederik Pauwels

**Affiliations:** 1 Infectious Diseases and Vaccines, Janssen Research and Development, Turnhoutseweg 30, 2340 Beerse, Belgium; nneto@its.jnj.com (N.C.-N.);; 2 Infectious Diseases and Vaccines, Janssen Research and Development, 1400 McKean Road, Springhouse, PA 19002, USA; 3 Infectious Diseases and Vaccines, Janssen Research and Development, 1600 Sierra Point Parkway, South San Fransisco, CA 94005, USA

**Keywords:** liver, T-cell exhaustion, sequencing, Trem2, hepatitis surface antigen

## Abstract

Background and Aims: Treatment with siRNAs that target HBV has demonstrated robust declines in HBV antigens. This effect is also observed in the AAV-HBV mouse model, which was used to investigate if two cycles of GalNAc-HBV-siRNA treatment could induce deeper declines in HBsAg levels or prevent rebound, and to provide insights into the liver immune microenvironment. Methods: C57Bl/6 mice were transduced with one of two different titers of AAV-HBV for 28 days, resulting in stable levels of HBsAg of about 10^3^ or 10^5^ IU/mL. Mice were treated for 12 weeks (four doses q3wk) per cycle with 3 mg/kg of siRNA-targeting HBV or an irrelevant sequence either once (single treatment) or twice (retreatment) with an 8-week treatment pause in between. Blood was collected to evaluate viral parameters. Nine weeks after the last treatment, liver samples were collected to perform phenotyping, bulk RNA-sequencing, and immunohistochemistry. Results: Independent of HBsAg baseline levels, treatment with HBV-siRNA induced a rapid decline in HBsAg levels, which then plateaued before gradually rebounding 12 weeks after treatment stopped. A second cycle of HBV-siRNA treatment induced a further decline in HBsAg levels in serum and the liver, reaching undetectable levels and preventing rebound when baseline levels were 10^3^ IU/mL. This was accompanied with a significant increase in inflammatory macrophages in the liver and significant upregulation of regulatory T-cells and T-cells expressing immune checkpoint receptors. Conclusions: Retreatment induced an additional decline in HBsAg levels, reaching undetectable levels when baseline HBsAg levels were 3log_10_ or less. This correlated with T-cell activation and upregulation of *Trem2*.

## 1. Introduction

Chronic infection with the hepatitis B virus (HBV, CHB) affects approximately 296 million individuals worldwide [1]. Currently approved therapies include chronic nucleos(t)ide analogues (NAs) and/or, less often, 48 to 96 weeks of pegylated interferon, which suppress viral replication [2,3]. However, the rate of functional cure (FC, defined as the sustained seroclearance of HBV DNA and the hepatitis B surface antigen (HBsAg) off treatment) is low. Pegylated interferon treatment for 1 year results in slightly higher rates of functional cure versus NAs but its well-known side effects can challenge patient compliance. NAs are well tolerated, but require long-term, often life-long, treatment [2,4]. Moreover, despite the effective suppression of viral replication and serum HBV DNA by NA treatment, which slows disease progression and prevents cirrhosis, there is a negligible effect on serum HBsAg concentrations, and the risk of hepatocellular carcinoma (HCC) remains significant due, at least in part, to the continued existence of hepatocytes with integrated HBV DNA [5]. Therefore, new treatment strategies are being investigated to achieve FC in CHB, thereby allowing treatment cessation and improving long-term clinical outcomes.

Multiple novel therapeutics for CHB, broadly classified as virus-targeting agents or immunomodulators, are being actively investigated and it is likely that a combination regimen with both types of compounds will be necessary to achieve FC [6]. RNA interference (RNAi), such as small interfering RNA (siRNA) [7,8] or antisense oligonucleotides (ASOs, [9]) can be designed to target the HBV, and both approaches show the ability to lower viral products including the HBsAg in clinical testing [6] (reviewed in [10]). RNAi, which uses homologous nucleotide strands to target post-transcriptional HBV mRNA which thus inhibit downstream viral protein production, can reduce HBsAg levels, regardless of baseline levels [11]. N-acetylgalactosamine (GalNAc) is used for liver-directing HBV-siRNA, as GalNAc binds to asialoglycoprotein receptors, a specific and abundant receptor in hepatocytes [12,13]. GalNAc-conjugation minimizes systemic siRNA exposure, reduces infusion reactions, lengthens dosing intervals, and enables administration through subcutaneous injection [12].

The HBV is characterized by four open reading frames (ORFs) that encode for HBV precore/core, polymerase, surface (envelope), and X proteins, respectively. The four ORFs have overlapping sequences and have the same polyadenylation signal at the 3′ end. This enables interference of all five HBV transcripts with a single siRNA, reducing all downstream viral proteins and pregenomic RNA production. Viral replication will therefore be reduced by siRNA, which may indirectly reduce the cccDNA reservoir [14]. siRNA is hence able to interfere with multiple steps of the viral lifecycle, directly or indirectly. Persistent infection with the HBV is thought to result from inadequate immune clearance of infected hepatocytes, specifically due to, at least in part, dysfunctional or depleted HBV specific CD4 and CD8 T-cells [15,16,17,18]. In CHB patients, T-cells have phenotypic, epigenetic, and functional features of exhaustion, with upregulated expression of immune checkpoint receptors such as the programmed death receptor 1 (PD-1) and T-cell immunoglobulin domain and mucin domain 3 (TIM3) [19]. Moreover, the liver microenvironment is inherently immunosuppressive, to protect against severe inflammation, and this can further impair the functionality of HBV-specific T-cells [18]. 

Treatment of CHB patients with siRNA and NA for 48 weeks showed a biphasic decline in HBsAg levels, plateauing at around week 16, and despite reducing HBsAg levels to up to 10 and 100 IU/mL, it did not lead to sustained HbsAg loss due to a rebound [20]. The biphasic kinetics and subsequent rebound of HbsAg levels after treatment seems to be a class effect [7,21].

This study conducted with the AAV-HBV mouse model aimed to determine whether a second cycle of HBV-siRNA treatment, administered after approximately 3 half-lives to ensure the availability of the RISC (RNA Induced Silencing Complex) [22,23], could prevent rebound and lead to sustained undetectable levels of HbsAg. Since HBV antigens have been reported to have a direct or indirect immune suppressive effect, [18,21] we wanted to investigate how the second treatment cycle may affect the liver’s immune microenvironment. Bulk RNA-sequencing was used to gain insights into those factors contributing to sustained undetectable levels of HbsAg. 

## 2. Materials and Methods

### 2.1. Ethical Statement and Animal Experimentation

Animal studies were conducted in strict accordance with guidelines established by the Janssen Pharmaceutica N.V. Institutional Animal Care and Use Committee and following the guidelines of the European Community Council directive of 24 November 1986 (Declaration of Helsinki 86/609/EEC). The local Johnson and Johnson Ethical Committee approved all experimental protocols performed at Janssen. The criteria of the American Chemical Society ethical guidelines for the publication of research were met. Every effort was made to minimize animal discomfort and to limit the number of animals used. Mice were kept in a specific pathogen-free facility under appropriate biosafety levels following institutional guidelines. 

### 2.2. Compounds

A GalNAc conjugated HBV siRNA and a control siRNA (siRNA encoding hAAT with GalNAc targeting moiety) were manufactured by Arrowhead Pharmaceuticals and Axolabs, respectively, for research purposes only. The GalNAc conjugated HBV siRNA is comprised of 2 short interfering RNAs (siRNAs) which target all HBV RNA transcripts derived from covalently closed circular DNA (cccDNA) and HBV DNA integrated into the host genome. siRNAs were conjugated with triantennerary N-acetylgalactosamine (GalNAc), facilitating hepatic delivery.

### 2.3. Animal Models and Study Designs

**Figure 1:**
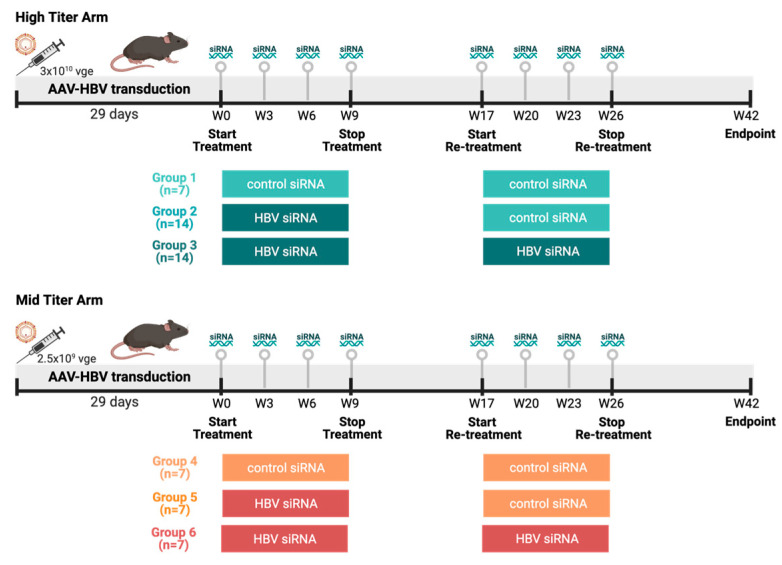
Study design overview. Female C57/Bl6 mice were transduced with different titers of AAV-HBV (High titer: 3 × 10^10^ vge, cold colors; Mid titer: 2.5 × 10^9^ vge, warm colors). After transduction (29 days), mice went through thefirst round of treatment either with a GalNAc-control-siRNA or a GalNAc-HBV-siRNA. After 8 weeks, a second round of treatment was administered either with a GalNAc-control-siRNA or a GalNAc-HBV-siRNA, and 16 weeks later endpoint samples were collected. The different groups described throughout the manuscript are labelled below. (Figure generated in Biorender).

Female C57BL/6 mice (6–8 weeks old, Janvier labs, Le Genest-Saint-Isle, France) were transduced with two viral genome equivalents of rAAV-HBV1.3-mer WT (Wild Type) replicon (genotype D, serotype ayw, BrainVTA, Wuhan, China) via the tail vein to obtain different HbsAg levels: the mid titer 2.5 × 10^9^ vge/mice and the high titer 3 × 10^10^ vge/mice. Blood sampling was performed 8 days before treatment to randomize treatment groups based on HbsAg levels. Treatment was initiated once stable HBV viremia was reached (day 28 after transduction). At day 28 after transduction, the high titer and mid titer mice were divided into three groups each (Figure 1). Group 1 and 4 (control) mice (n = 14) were treated twice subcutaneously with 3 mg/kg control siRNA 4 times on a 3-week basis, with a treatment pause of 8 weeks in between. Group 2 and group 5 (single treated) mice (n = 21) were treated with HBV siRNA 3 mg/kg 4 times on 3-week basis; after 8 weeks treatment pause these were retreated with 3 mg/kg control-siRNA 4 times on a 3-week basis. Group 3 and group 6 (retreated mice) mice (n = 21) were treated twice with HBV-siRNA 4 times on a 3-week basis with a treatment pause of 8 weeks in between. A power analysis on historical data was performed to determine the number of animals needed in each group to reach meaningful results.

Blood for viral parameters was collected via the lateral saphenous vein every week, from which serum was prepared and stored at −80 °C until assayed. At the end of the study the liver was formalin fixed and embedded in paraffin to determine HBs and HB core antigen levels. Liver resident T-cells were also collected to perform bulk sequencing. 

### 2.4. Viral Parameters and Alanine Aminotransferase (ALT) Analyses

Serum HbsAg, HbeAg, and anti-HBs levels were quantified using CLIA kits (Ig Biotechnology, Burlingame, CA, USA). All CLIA kits were used according to the manufacturer’s guidance. Depending on the estimated levels, different dilutions of serum in PBS (Phosphate Buffered Saline) were used. Read-out of plates was performed with a Viewlux ultra HTS (High-throughput screening (HTS) microplate imager (Perkin Elmer, Mechelen, Belgium).

The serum alanine aminotransferase (ALT) activity was analyzed using a commercially available kit according to the manufacturer’s guidance (Sigma-Aldrich, Saint Louis, MO, USA)), and a Spark multimode microplate reader (Tecan, Mechelen, Belgium). A total of 4 µL of serum was used to perform the assay. 

### 2.5. Histology and Immunohistochemistry (IHC)

Formalin-fixed paraffin-embedded livers were sectioned at 5µm thickness, mounted on pre-charged slides, and stained with HbcAg (Dako, Glostrup, Denmark) and HbsAg (Abcam, Cambridge, UK). Tissues were subjected to an EDTA-based antigen retrieval, while signal amplification and detection was achieved with a hapten multimer and a DAB chromogenic detection kit, on a Ventana Discovery Ultra autostainer (Roche Diagnostics, Rotkreuz, Switzerland). Negative control staining was performed for every antibody using an isotype control (Rabbit PE IgG for HbcAg, mouse IgG1 for HbsAg, Table S1). Sections were coverslipped with an automated Ventana HE600 (Roche Diagnostics, Rotkreuz, Switzerland) and scanned using a Hamamatsu Nanozoomer RX.

### 2.6. Image Analysis

IHC stainings were evaluated using the HALO^TM^ system (Halo 3.4). Liver tissue was discriminated from glass/lumen by using a tailored random forest classifier in HALO v3.4. and expressions of HbcAg and HbsAg were evaluated using two tailored algorithms based on nuclear or signal area detection, respectively. Output parameters included percentage of HbcAg-positive cells, relative to total number of haematoxylin-positive cells, and HbsAg-positive surface area, relative to the total classified liver area analyzed. 

### 2.7. Isolation Intrahepatic Lymphocytes (IHLs)

IHLs were obtained by perfusing the liver with PBS via the hepatic portal vein to flush out excess blood. Ex vivo perfusion, enzymatic digestion, and tissue dissociation of the left lateral liver lobe were performed using a GentleMACS Octo Dissociator with heaters and the liver perfusion kit for mice according to the manufacturer’s protocol (Miltenyi Biotec, Gladbach, Germany). Hepatocytes were separated from lymphocytes by centrifugation at 50× *g* for 5 min. Supernatant was spun down at 400× *g* for 5 min followed by resuspension in 33.75% (*v*/*v*) Percoll (GE Healthcare) diluted in PBS with 2% fetal calf serum and density gradient centrifugation at 700× *g* for 12 min. Next, residual hepatocytes and debris were discarded, and red blood cells co-sedimented with intrahepatic immune cells (IHICs) were lysed using ACK lysis buffer (Lonza, Basel, Switzerland) for 5 min. Cells were washed twice and counted. Cell concentration and viability were determined using a Nexcelom Cellaca MX Cell Counter. 

### 2.8. Bulk Liver RNA-Sequencing and Data Analysis

Total RNA was extracted using a Qiagen Rneasy Plus Micro kit following manufacturer’s instructions. RNA samples were quantified using a Qubit 4.0 Fluorometer (Life Technologies, Carlsbad, CA, USA) and RNA integrity was checked with an RNA Kit on an Agilent 5300 Fragment Analyzer (Agilent Technologies, Palo Alto, CA, USA). 

rRNA depletion was performed using an NEBNext rRNA Depletion Kit (Human/Mouse/Rat). RNA sequencing library preparation was performed using an NEBNext Ultra II RNA Library Prep Kit for Illumina by following the manufacturer’s recommendations (NEB, Ipswich, MA, USA). Briefly, enriched RNAs were fragmented according to manufacturer’s instruction. First strand and second strand cDNA were subsequently synthesized. cDNA fragments were end repaired and adenylated at 3′ends, and a universal adapter was ligated to cDNA fragments, followed by index addition and library enrichment with limited cycle PCR. Sequencing libraries were validated using an NGS Kit on an Agilent 5300 Fragment Analyzer (Agilent Technologies, Palo Alto, CA, USA), and quantified using an Qubit 4.0 Fluorometer (Invitrogen, Carlsbad, CA, USA). 

The sequencing libraries were multiplexed and loaded on the flow cell on an Illumina NovaSeq 6000 instrument according to manufacturer’s instructions. The samples were sequenced using a 2 × 150 Pair-End (PE) configuration v1.5. Image analysis and base calling were conducted by the NovaSeq Control Software v1.7 on the NovaSeq instrument. Raw sequence data (.bcl files) generated from Illumina NovaSeq were converted into fastq files and de-multiplexed using the Illumina bcl2fastq program version 2.20. One mismatch was allowed for index sequence identification. Fastq files were filtered for quality and adapters using fastp. Mapping to the mouse genome (mm10) was performed using STAR (v2.7) using standard parameters. For RNAseq counts data, we obtained raw counts and filtered out low or no-expressing genes using edgeR’s filterbyExpr function using a design matrix constructed from the treatment contrasts of the samples. Subsequent to this, we performed library normalization, dispersion estimation and differential gene expression using edgeR’s glmQLFit. 

### 2.9. Flow Cytometry 

All staining procedures were performed for 30 min at 4 °C and all washing steps were conducted at 400× *g* for 5 min at 4 °C. Cells were treated with True stain monocyte blocker (Biolegend) and an anti-CD16/CD32 FC blocking reagent (clone 2.4G2, BD) for 10 min, after which a dead cell exclusion marker (fixable viability dye eFluor780, Invitrogen) was co-incubated for 30 min. After washing the cells with stain buffer, staining was performed on ice using the following panel of fluorochrome-conjugated antibodies diluted in stain buffer (0.5% BSA (Bovine Serum Albumin) supplemented with brilliant stain buffer plus (BD)): CD45-BUV395 (clone 30-F11), CD8a-BUV496 (Clone 53–6.7), CD4-BV786 (clone GK1.5), CD3-PerCP-Cy5.5 (Clone 17A2), PD1-BV605 (clone J43), LAG3-BUV737 (clone C9B7W), TIM3-BB515 (clone 5D12), (BD) and TIGIT-PE (cloneA17200C), and CD154-PE-Cy7 (clone MR1, all mAbs from BioLegend, Table S2). After staining of the cell surface proteins, cells were washed twice with stain buffer, fixed with a fixation reagent (Invitrogen) and permeabilized twice using a Foxp3/Transcription Factor Staining buffer set (Invitrogen) before being stained on ice with a second panel of antibodies added to diluted permeabilization buffer (BD) as follows: TOX-A647 (cloneNAN448B, BD), Foxp3-PE-CF594 (Clone 3G3, BD), and TCF1-A405 (clone #812145, RnD systems). Finally, cells were washed twice with permeabilization buffer and left in 200 µL of Stain buffer BSA in the dark at 4 °C until cytometry data were acquired on a BD LSRFortessa instrument. Data were analyzed using FlowJo (BD). The following gating strategy was followed to determine pre-exhausted, exhausted, and terminally exhausted T-cells (Figure S1): Lymphocyte gate was made based on forward scatter (FSC)-side scatter (SSC). Next, single cells were selected based on FSC-Area/SSC-Area. Live dead stain was plotted against CD45 to select the living CD45+ cells. Next, SSC was plotted against CD3 to select T-cells. CD4 was plotted against CD8 within the CD3+ T-cell gate to distinguish between CD8 and CD4 T-cells. In both these gates, the expression of PD-1, TOX, TIM-3, LAG-3, and TIGIT was evaluated. Tregs were characterized as FOXp3+ within the CD4 T-cells. 

### 2.10. Statistical Analysis

Statistical comparisons were performed using a GrapPad Prism 9. To compare statistical differences between distinct groups, a one-way ANOVA was used, and *p*-values are indicated in the respective figures. To evaluate declines in HbsAg levels, simple linear regression was performed.

## 3. Results

### 3.1. Two Cycles (But Not One) of HBV-siRNA Treatment Achieve Undetectable HbsAg and HbeAg Levels in a Baseline HbsAg Level Dependent Manner

**Figure 2:**
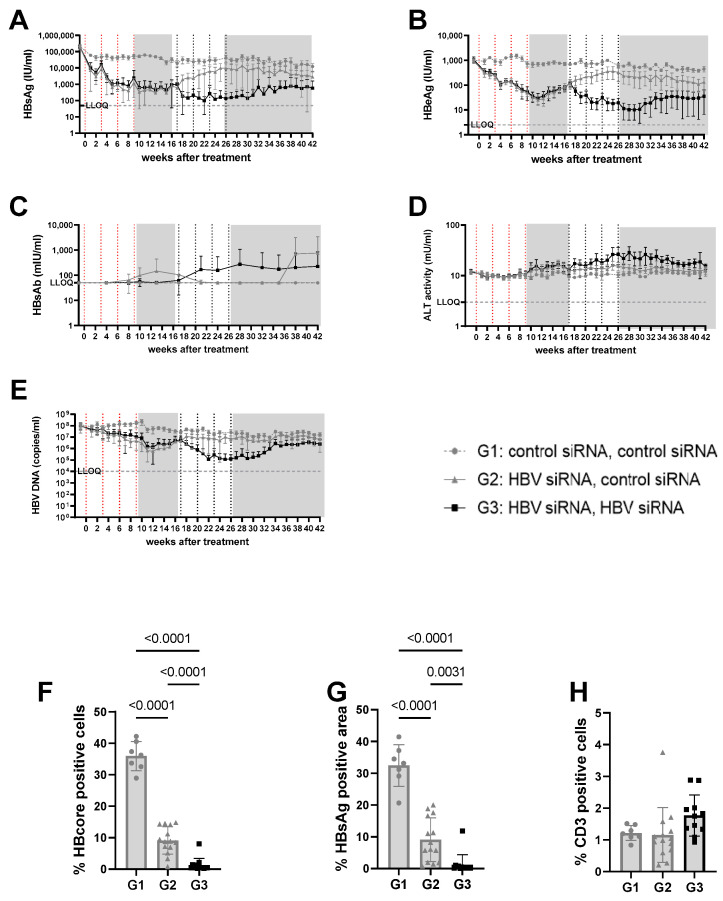
Viral parameters in serum and liver of mice transduced with high titer virus. High titer mice were mice transduced with 3 × 10^10^ vge/mice of AAV-HBV. Twenty-nine days after transduction, treatment was started. The first treatment cycle of 4 doses of 3 mg/kg siRNA every 3 weeks is indicated with red dotted lines. The second treatment cycle is indicated with black dotted lines. The treatment free periods are indicated in grey boxes. G1 received two treatments with GalNAc-control-siRNA (grey rounds). G2 received GalNAc-HBV-siRNA first and then GalNAc-control-siRNA (grey triangle). G3 received GalNAc-HBV-siRNA (black squares) twice. Blood was taken on a weekly basis to evaluate viral parameters. (**A**) HbsAg levels, (**B**) BeAg levels, (**C**) HBs antibodies, (**D**) ALT levels, (**E**) viral load. At the end of the study, liver was paraffin fixed and HB core Ag (**F**), HBsAg levels (**G**), and CD3 infiltration (**H**) were evaluated.

**Figure 3:**
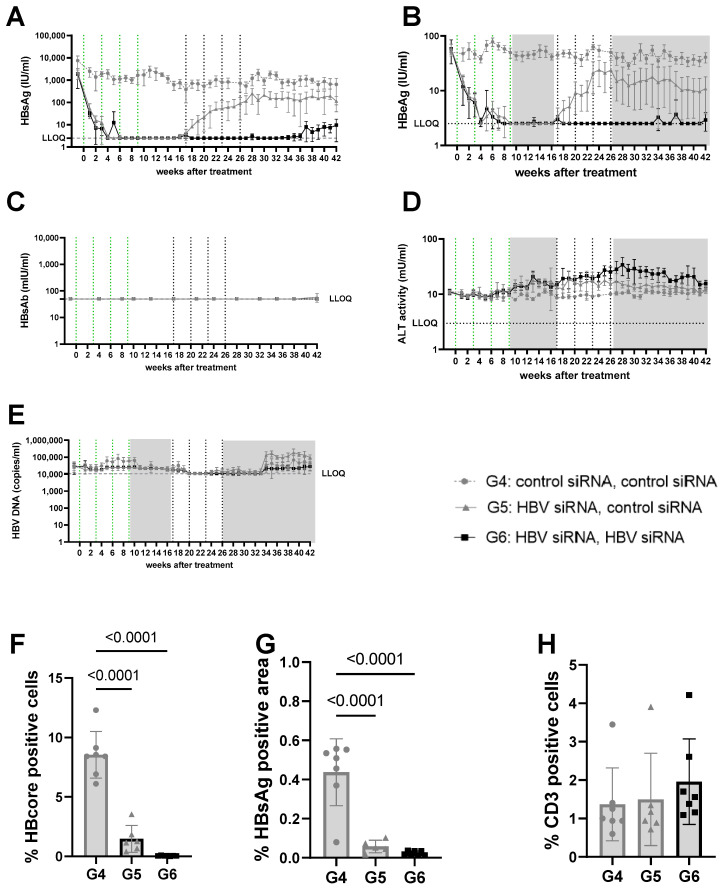
Viral parameters in serum and liver of mice transduced with mid titer virus. Mid titer mice were mice transduced with 2.5 × 10^9^ vge/mice of AAV-HBV. Twenty-nine days after transduction, treatment was started. The first treatment cycle of 4 doses of 3 mg/kg siRNA every 3 weeks is indicated with green dotted lines. The second treatment cycle is indicated with black dotted lines. The treatment free periods are indicated in grey boxes. G4 received two treatments with GalNAc-control-siRNA (grey rounds). G5 received GalNAc-HBV-siRNA first and then GalNAc-control-siRNA (grey triangle). G6 received GalNAc-HBV-siRNA (black squares) twice. Blood was taken on a weekly basis to evaluate viral parameters. (**A**) HBsAg levels, (**B**) HBeAg levels, (**C**) HBs antibodies, (**D**) ALT levels, (**E**) viral load. At the end of the study, liver was paraffin fixed and HB core Ag (**F**), HBsAg levels (**G**), and CD3 infiltration (**H**) were evaluated.

As demonstrated before, the administration of HBV-siRNA to AAV-HBV mice with stable 5log_10_ (high) or 3log_10_ (mid titer) HbsAg levels (Figure 1) achieved a significant reduction in viral parameters (HbsAg, HbeAg, HBV DNA, Figure 2A–E, Figure 3A–E and Figure S2), but this was not seen in groups of mice receiving the control siRNA (Figure 2A,B, Figure 3A,B, Figures S3 and S6).

n the high titer arm (5log_10_ IU/mL baseline HBsAg concentrations, groups 1–3), one cycle of HBV-siRNA treatment (groups 2 and 3) reduced HBsAg levels by 2 to 3log_10_*,* reaching a plateau at 2.6log_10_ IU/mL by week 5 that was maintained for 10 weeks, followed by a gradual rebound starting at week 17 after the start of treatment (Figures S4 and S5). Retreatment with a second cycle of HBV-siRNA (G3) prevented the rebound and resulted in an additional 1log_10_ reduction in HBsAg levels that followed similar biphasic kinetics, with a plateau at 2log_10_ IU/mL HBsAg. Although the magnitude of the decline was less than after the first cycle (1log_10_), by the end of the second cycle the retreated mice (G3) had HBsAg levels of 1log_10_ or 2log_10_ lower than the single treated (G2) or control (G1) mice, respectively (4log_10_ HBsAg for G1 versus 3log_10_ for G2 and 2log_10_ for G3, *p* < 0.0001). HBeAg and DNA levels both followed a similar pattern as the HBsAg levels (Figure 2B,E and Figure S2). 

At a lower (3log_10_ IU/mL) baseline HBsAg level (mid titer groups, G4–6) a single cycle of HBV-siRNA (G5 and G6) reduced HBsAg levels to a similar extent as described above and with similar slope and kinetics, but achieved undetectable levels of both HBsAg and HBeAg by week 6, which rebounded by week 17 (Figure 3A,B, Figures S7 and S9A). A second cycle of treatment (G6), however, maintained HBsAg and HBeAg undetectable levels (<2.5 IU/mL) for the HBeAg until the end of the study (week 42), whereas a slight rebound of the HBsAg levels was observed by week 32 to approximately 0.8log_10_ IU/mL (Figure 3 and Figure S8B). At the end of the study, HBsAg levels were 2.6log_10_ (G4), 1.9log_10_ (G5), and 0.8log_10_ (G6), which is >2log_10_ below baseline (3log_10_ versus 0.8log_10_), with one out of seven mice remaining below the detection limit after retreatment (Figure S8A). Anti-HBs were not induced in mice treated with the control siRNA and only rarely in the groups that received HBV-siRNA (Figure 2C, Figure 3C, Figures S4 and S5), suggesting that the induction of antibodies is not the driver of the reduction in HBsAg levels. 

ALT was measured to evaluate whether the reduction in HBsAg levels was due to the elimination of infected hepatocytes through immune reconstitution (Figure 2D and Figure 3D). No ALT flares were observed in the control (G1, G4) or single treated (G2, G5) groups of mice (Figure 2D, Figure 3D, Figures S3E, S4E, S6E and S7E). However, all mice except for one in the retreated groups (G3, G6) showed moderate ALT flares, mainly during the second treatment cycle (Figure 2D, Figure 3D, Figures S5E and S8E). While not all mice with flares achieved sustained low HBsAg levels, the one mouse that did not have elevated ALT had the highest HBsAg levels (Figure S5E).

Given the observed peripheral reduction in viral parameters, an important aspect of this study was to understand whether this also influenced the liver microenvironment. At the end of the study, intrahepatic HBV core antigen (HBcAg) and HBsAg levels were significantly lower after two cycles of treatment (G3) than after only one cycle (G2) (*p* < 0.0001 for core; *p* = 0.0031 for S) or in the control mice (G1) (*p* < 0.0001, for HBcAg Figure 2F; *p* < 0.0001 for S, Figure 2G). This was similar when baseline levels were lower (mid titer group); compared to the control (G4) groups, intrahepatic HBsAg levels were significantly lower after one treatment cycle (G5) (*p* < 0.0001 for core and s, Figure 3F,G) and retreatment (G6) reduced levels further (*p* < 0.0001 for core and s; Figure 3F,G).

### 3.2. Bulk RNA-Sequencing Shows Modest Changes in the Liver after siRNA Treatment

To understand whether the efficacy observed after two treatment cycles was the result of changes in the liver microenvironment, intrahepatic bulk RNA-sequencing was performed at the end of the study (16 weeks post retreatment, week 42; Table 1). Baseline HBsAg levels did not influence gene expression in the control groups (G1, n = 6, G4, n = 5) (Figure S10), suggesting that both baseline levels of HBsAg were similarly immune suppressive.

**Figure 4:**
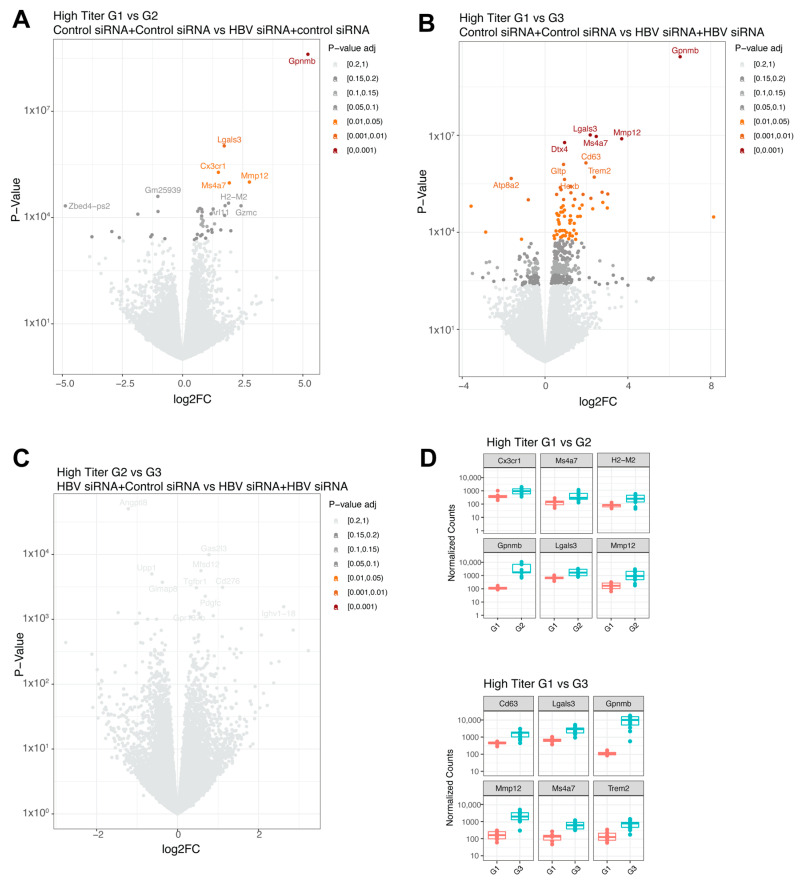
Differential expressed genes in high titer transduced mice groups. (**A**) Volcano plot with the comparison between group 1 (GalNAc-control-siRNA + GalNAc-control-siRNA) and group 2 (GalNAc-HBV-siRNA + GalNAc-control-siRNA). (**B**) Volcano plot with the comparison between group 1 (GalNAc-control-siRNA + GalNAc-control-siRNA) and group 3 (GalNAc-HBV-siRNA + GalNAc-HBV-siRNA). (**C**) Volcano plot with the comparison between group 2 (GalNAc-HBV-siRNA + GalNAc-control-siRNA) and group 3 (GalNAc-HBV-siRNA + GalNAc-HBV-siRNA). Colors represent fdr adjusted *p*-values. (**D**) Boxplots with normalized counts of the top 6 significant genes per contrast. In red results from group 1, in green results of group 3.

**Figure 5:**
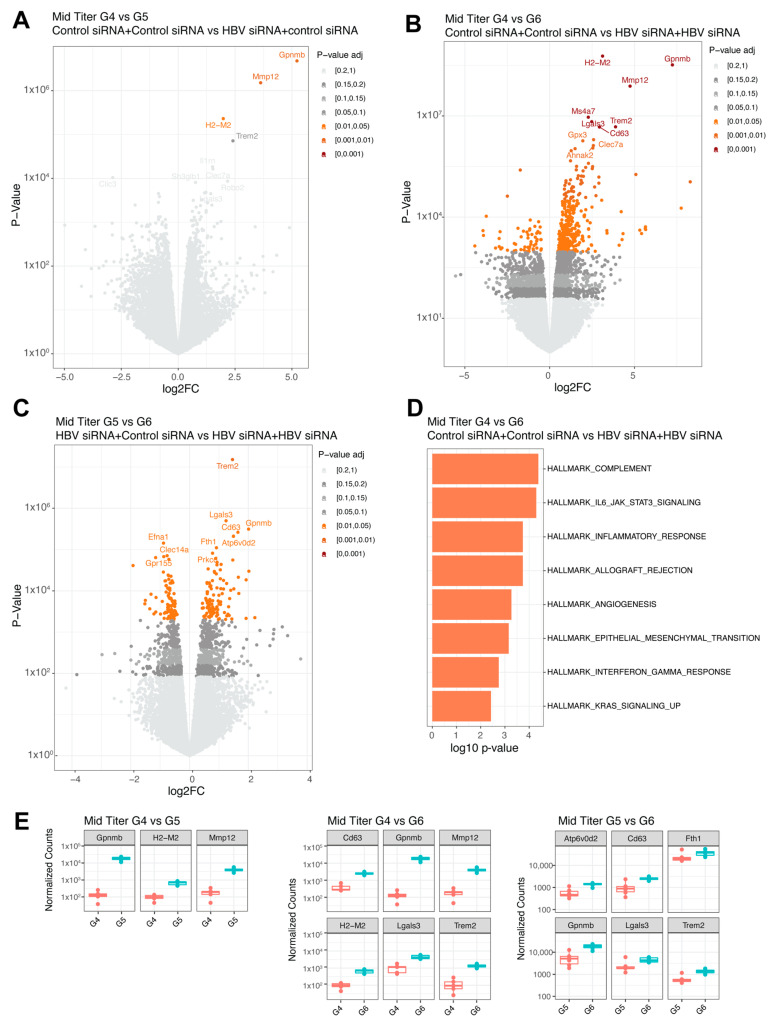
Differential expressed genes in mid titer transduced mice groups. (**A**) Volcano plot with the comparison between G4 (GalNAc-control-siRNA + GalNAc-control-siRNA) and G5 (GalNAc-HBV-siRNA + GalNAc-control-siRNA). (**B**) Volcano plot with the comparison between G4 (GalNAc-control-siRNA + GalNAc-control-siRNA) and G6 (GalNAc-HBV-siRNA + GalNAc-HBV-siRNA). (**C**) Volcano plot with the comparison between G5 (GalNAc-HBV-siRNA + GalNAc-control-siRNA) and G6 (GalNAc-HBV-siRNA + GalNAc-HBV-siRNA). Colors represent fdr adjusted *p*-values. (**D**) Pathway enrichment analysis lot with the comparison between G4 and G6 using the Hallmark 50 pathways. (**E**) Boxplots with normalized counts of the top significant genes per contrast. The different colors indicate different groups.

In the baseline high titer groups, a single treatment (G2, n = 13) significantly upregulated five genes (*Gpnmb*, *Lgals3*, *Cx3cr1*, *Mmp12*, and *Ms4a7*, shown in decreasing order of significance; Figure 4A and Figure 5D) compared to the control group (G1, n = 6). When comparing the control group (G1, n = 6) with the retreatment group (G3, n = 12), 72 differentially expressed genes were identified (Figure 4B and Figure 5D). The top upregulated genes were *Gpnmb, Lgals3, Mmp12, Dtx4*, and *Ms4a7*, which largely overlapped with those observed after a single treatment. No significant differences were found between the single treated (G2, n = 13) and retreated groups (G3, n = 12) (Figure 4C).

In the mid titer groups, three genes were significantly upregulated (*Gpnmb, Mmp12*, and *H2-M2,* Figure 5A,E) when comparing the control group (G4) to the single treatment group (G5). A total of 425 genes were differentially expressed when compared to the retreatment group (G6, n = 6), with the highest fold changes (>3log) shown for *H2-M2, Gpnmb*, *Mmp12*, and *Trem2* (Figure 5B,E) and increased inflammatory responses (complement and IL-6, Figure 5D) showed by pathway enrichment. Comparing the single treatment group (G5, n = 6) to the retreatment group (G6, n = 6) yielded differential expression for 196 genes (Figure 5C), with *Trem2* showing the greatest upregulation in the retreated group (Figure 5E), followed by *Gpnmb* and *Lgals3*. Overall, across the different comparisons, the same genes were upregulated.

### 3.3. HBV-siRNA Retreatment Enhances Expression of Checkpoint Receptors and Upregulates CD4 Regulatory T-Cells (Treg) Compared to Control Mice

At the end of the study, CD3 T-cell infiltration was evaluated to see if immune reconstitution might correlate with higher virological responses (Figure 2H). While levels of CD3 T-cells were low overall, and differences were not significant, there appeared to be a trend for more CD3 T-cells in the liver after retreatment (G3) compared to the single treatment (G2) or control groups (G1, Figure 3H).

**Figure 6:**
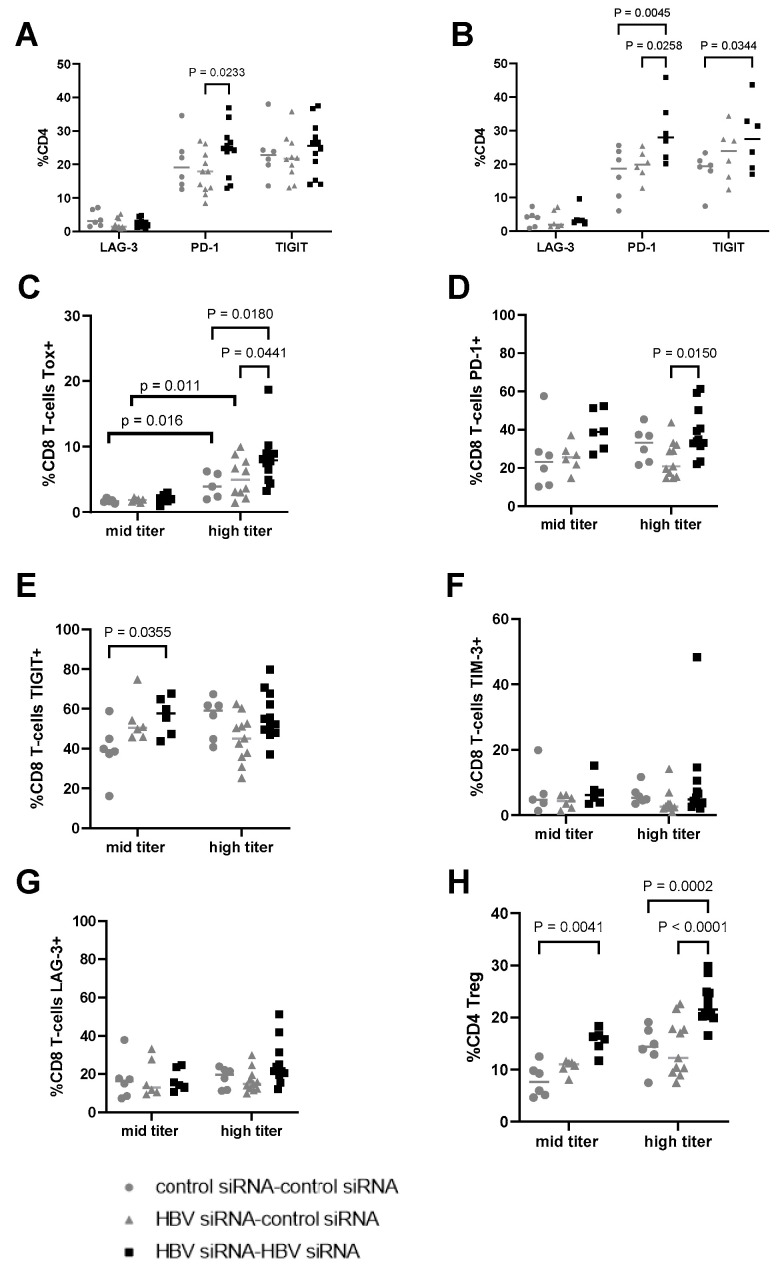
Evaluation of expression of checkpoint receptors on CD4 and CD8 T-cells in liver. (**A**) Percentage of CD4 T-cells expressing LAG-3, PD-1, and TIGIT in the different treatment groups, transduced with a high titer of AAV-HBV (3 × 10^10^ vge) and (**B**) transduced with a mid titer of AAV-HBV (2.5 × 10^9^ vge). Percentage of CD8 T-cells expressing (**C**) TOX, (**D**) PD-1, (**E**) TIGIT, (**F**) TIM-3, and (**G**) LAG-3. (**H**) Percentage of T-regs in total CD4 (characterized as CD4+, FOXP3+). As statistical analysis, a non-parametric Wilcoxon test with fdr correction was performed; *p*-values are indicated on the graph. G1 and G4 received two GalNAc-control-siRNA treatments (grey rounds). G2 and G5 received GalNAc-HBV-siRNA first and then GalNAc-control-siRNA (grey triangle). G3 and G6 received GalNAc-HBV-siRNA (black squares) twice.

Intrahepatic lymphocytes were isolated at the end of the study and characterized to evaluate the frequency of immune suppressive T-cells by determining the expression of checkpoint receptors on CD4 and CD8 T-cells (Figure 6A–G). The expression of checkpoint receptors on CD4 T-cells was comparable regardless of baseline HBsAg titer in the control groups. Interestingly, PD-1 and TIGIT were significantly upregulated on CD4+ T-cells after retreatment but not after a single treatment cycle (Figure 6A,B).

On CD8 T-cells, the expression levels of checkpoint receptors were greater in the high titer groups compared to the mid titer groups. TOX expression in HBV-specific CD8 T-cells has been linked to chronic antigen stimulation, correlated with viral load and associated with phenotypic and functional characteristics of T-cell exhaustion [24]. The expression levels of TOX were significantly higher (*p* < 0.05) in the high titer groups (G4–G6) compared to the mid titer ones (G1, G2, G3; Figure 6C). Interestingly, in the high titer groups, additional differences were observed for TOX, with levels higher after retreatment (G6, black squares) vs. single (G5, grey triangles, *p* = 0.0441) or no treatment (G4, grey circles, *p* = 0.018). Expression levels of PD-1 and TIGIT were also higher in the high titer groups compared to mid titer for the control groups (grey circles, Figure 6D,E). Higher expression of PD-1, and TIGIT on the CD8 T-cells compared to the control group in both high and mid titer mice, while the expression of TIM-3 and LAG-3 was comparable across groups (6F–G) after retreatment with HBV-siRNA. 

The evaluation of CD4 Tregs showed that their frequency was greater in the groups with a higher baseline level of HBsAg (Figure 6H). Regardless of the baseline titer, upon retreatment with HBV-siRNA an increase in the frequency of CD4 Treg cells was observed compared to those mock treated (*p* = 0.041 for the mid titer and *p* < 0.0001 in high titer), indicating that T-cells are activated in the retreatment arms and subsequently become exhausted given the continuous antigen stimulation as HBsAg levels remain high during the treatment phase. 

## 4. Discussion

A finite treatment for CHB that delivers functional cure is expected to significantly improve HBV-related morbidity and mortality, mostly by reducing the risk of developing HCC, while also removing the stigma associated with HBV infection [25]. To this effect, numerous therapeutic approaches are being investigated, both at preclinical and clinical stages, with a key objective being to reduce immune suppressive HBV antigens, particularly the HBsAg, to allow the restoration of HBV-specific immune control, through a variety of mechanisms of action and therapeutic modalities [10,26]. 

Small interfering RNA (siRNA) is one approach that has demonstrated clinical efficacy, with rapid reductions in HBsAg levels [7,8,9,20,27,28,29,30,31]. CHB patients treated with HBV-siRNA and NA for 48 weeks experienced sustained HBsAg reductions of at least 2 logs for one year after the end of treatment in a considerable proportion of patients, that might suggest some recovery of immune function [20]. This decline shows biphasic kinetics, with HBsAg levels plateauing at around week 12–20, and although levels of HBsAg are reduced significantly, functional cure is rarely achieved due to the characteristic rebound after treatment. Additionally, given the long pharmacodynamic activity of siRNA molecules, multiple dosing regimens of HBV-siRNA explored in the clinic have been shown to provide comparable but not improved reduction in HBsAg levels, with the suggestion that continuous RISC loading could lead to its saturation being a reason behind this effect [8,20,21]. 

Using the AAV-HBV mouse model, we investigated administering a second cycle of GalNAc-HBV-siRNA after an 8-week pause following the first treatment cycle. This was approximately 3 half-lives to allow for a “wash-out” period where the RISC is assumed to be free again in order to load new siRNA molecules [22,23]. This could potentially increase the effectiveness of the treatment and possibly allow for the development of a stronger HBV-specific immune response. 

This study showed that two treatment cycles with a GalNAc-HBV-siRNA drove a further reduction in HBsAg levels in serum and in the liver compared to a single treatment. In the high titer arm, at the end of the study 43% of mice (6/14) showed a >2log_10_ reduction in HBsAg levels in serum compared to the baseline after a single HBV-siRNA cycle, whereas this was observed in 93% of mice (13/14) that received two cycles of HBV-siRNA. When baseline HBsAg levels were lower (3log_10_ IU/mL, mid titer groups), the second cycle of siRNA brought HBeAg (7/7, 100%) and HBsAg levels (5/7, 71%) below detectable concentrations for 38 and 36 weeks, respectively. 

While the kinetics of HBsAg reduction were similar during the second treatment, it prevented the characteristic post treatment rebound 17 weeks following the first treatment. It is important to note that the second cycle achieved a smaller relative HBsAg reduction (approximately 1log_10_) compared to the first treatment cycle, where it was approximately 2log_10_, but also had less rebound by week 42. 

The observed mild increase in ALT and the activation of the myeloid compartment upon second treatment support that this additional reduction in HBsAg levels is due to the active elimination of infected hepatocytes by the immune system, and not the result of hepatocyte turnover in the AAV-HBV model, which does not support reinfection. 

In the context of a lower level of baseline antigenemia, (mid titer, G6), where antigen levels were reduced to undetectable for a substantial number of weeks, a lower level of checkpoint receptors were observed, likely a reflection on the reduced antigen activation environment. However, in the high titer mice (G3), which did not reach sustained HBsAg loss, an upregulation of TOX was observed. TOX is believed to be a biomarker of dysfunctional virus specific CD8 T-cells [24]. Due to the upregulation of TOX, it can be speculated that upon initial activation, these T-cells become more exhausted compared to those in the mid titer mice, therefore preventing a sustained viral control. 

It is hypothesized that high concentrations of HBsAg contribute to T- and B-cell dysfunction [32,33,34,35,36,37,38,39,40]. This immune exhaustion impairs the host’s ability to eradicate or control the HBV infection, since these T-cells upregulate different inhibitory receptors [32,33,35]. In this study, we show that after retreatment, in both the high and mid titer groups, significantly higher numbers of CD4 Tregs were detected upon retreatment. Moreover, TIGIT and PD-1 were upregulated in both CD4 and CD8 T-cells. PD-1 and TIGIT can be seen as either activation or exhaustion markers [19]. High HBsAg levels drive the significantly increased frequency of CD8 T-cells in the liver expressing TOX, with a trend towards upregulation of PD-1 and TIGIT expression. The frequency of CD4 Tregs was also upregulated in mice with higher HBsAg levels compared to those with lower HBsAg levels (comparing G1, 5log_10_ HBsAg with G4, 3log_10_ HBsAg). This all points to more exhausted T-cells that could become dysfunctional and immunosuppressive in the presence of higher levels of viral antigens. We hypothesize that T-cells are activated due to decreasing HBV antigenemia upon retreatment and could play a role in the observed reduction in HBsAg and HBeAg levels. 

In the high titer baseline group where HBsAg levels were still at/above 2log_10_ IU/mL after retreatment, significantly more TOX+ CD8+ T-cells, compared to low titer mice, were detected. Lower levels of phenotypic markers of exhaustion were observed when HBsAg levels remained undetectable for a longer duration (mid titer group 6), suggesting retreatment brought a certain level of immune reconstitution or recovery with the ability to control the infection. However, due to the competition between additional HBsAg production and activated T-cell driven control, we speculate that eventually T-cells get exhausted, resulting in a slight increase in HBsAg levels to (0.8log_10_ IU/mL) in the absence of additional treatment. 

Additionally, at the transcriptome level, the liver microenvironment was comparable regardless of the baseline HBsAg levels. With regards to the liver immune microenvironment, we show that only in mice that reached undetectable or low HBsAg levels (0.8log_10_ +/−0.13HBsAg), a more activated liver immune compartment was found (G5 vs. G6, 196 DEG) after retreatment. This was not observed in the high titer group, where mean HBsAg levels remained ≥ 2log_10_ (G2 vs. G3, 0 DEG). 

Interestingly, *Trem2* was shown to be significantly increased in the retreated groups versus the single or no treatment control groups in mid titer mice. Hendrikx and colleagues have described that TREM2+ macrophages localize to fibrotic areas and limit NASH (Non Alcoholic Steato Hepatitis) severity [41]. In the same NASH study, the absence of TREM2+ macrophages was accompanied by a decrease in *Gnpmb*, *Cd63*, *Mmp12*, and *Lgals3* expression. Another study in acute and chronic hepatotoxic injury in mice observed that *Trem2* was responsible for promoting the transition from the pro-inflammatory phase to tissue repair phase in macrophages [42]. We speculate that the upregulation of *Trem2* in the liver after siRNA (re)treatment is due to recruitment of anti-fibrotic TREM2+ macrophages. This could suggest that siRNA treatment induces repair mechanisms in the liver that are further increased upon retreatment. However, this study performed bulk RNA profiling and not single-cell RNA-sequencing, which would be able to confirm an expansion of *Trem2+* macrophages in the liver after retreatment.

In conclusion, our findings from the AAV-HBV mouse model demonstrate that administering a second cycle of GalNAc-HBV-siRNA, following a pause to allow for the elimination of the siRNA from the RISC, leads to a significant additional decrease in HBs- and HBeAgs concentrations in serum and in the liver. Importantly, this reduction occurs regardless of the initial HBsAg levels prior to treatment. One limitation of this study is the lack of a continuous 42-week siRNA treatment arm. Clinical data with GalNAc siRNA show a plateauing of the HBsAg decline after about 16–20 weeks [7,8,10,12,20,21,27,31], and data from Yuen et al. [21] suggest that the long pharmacodynamic activity of siRNA requires a dosing interval long enough to allow the RISC to be free to upload new siRNA molecules. Alternatively, following a cycle of GalNAc-HBV-siRNA with a RISC-independent HBsAg-lowering treatment, for example an ASO, could lead to a sustained reduction in HBsAg levels and tilt the dynamic interplay between reducing viral parameters and activating the immune response, towards functional cure. 

## Figures and Tables

**Table 1:** DEG across the different bulk RNA-seq comparisons.

Group A	Group B	# Significant Genes (Adjusted *p*-Value < 0.05)
G1 High titer control: Control siRNA + Control siRNA (n = 6)	G4 Mid titer control: Control siRNA + Control siRNA (n = 5)	0
G1 High titer control: Control siRNA + Control siRNA (n = 6)	G2 High titer single treatment: Control siRNA + HBV siRNA (n = 13)	5
G1 High titer control: Control siRNA + Control siRNA (n = 6)	G3 High titer retreatment: HBV siRNA + HBV siRNA (n = 12)	72
G2 High titer single treatment: Control siRNA + HBV siRNA (n = 13)	G3 High titer retreatment: HBV siRNA + HBV siRNA (n = 12)	0
G4 Mid titer control: Control siRNA + Control siRNA (n = 5)	G5 Mid titer single treatment: Control siRNA + HBV siRNA (n = 6)	3
G4 Mid titer control: Control siRNA + Control siRNA (n = 5)	G6 Mid titer retreatment: HBV siRNA + HBV siRNA (n = 6)	425
G5 Mid titer single treatment: Control siRNA + HBV siRNA (n = 6)	G6 Mid titer retreatment: HBV siRNA + HBV siRNA (n = 6)	196

## Data Availability

The raw counts and metadata from the bulk RNA-seq analysis are available in Zenodo: 10.5281/zenodo.10696666.

## Supplementary Materials

The following supporting information can be downloaded at: https://www.mdpi.com/article/10.3390/v16030347/s1, Figure S1: T-cell gating; Figure S2: HBV DNA in serum of mice transduced with AAV-HBV and treated with GalNAc HBV siRNA; Figure S3:Viral parameters in serum and liver of mice transduced with a high titer virus and treated twice with control GalNac-siRNA; Figure S4: Viral parameters in serum and liver of mice transduced with a high titer virus and treated with GalNac-HBV-siRNA and control GalNac-siRNA; Figure S5: Viral parameters in serum and liver of mice transduced with a high titer virus and treated twice with GalNac-HBV-siRNA; Figure S6: Viral parameters in serum and liver of mice transduced with a mid titer virus and treated twice with control GalNac-siRNA; Figure S7: Viral parameters in serum and liver of mice transduced with a mid titer virus and treated with GalNac-HBV-siRNA and control GalNac-siRNA; Figure S8: Viral parameters in serum and liver of mice transduced with a high titer virus and treated twice with GalNac-HBV-siRNA; Figure S9: changes from baseline in HBsAg levels during first and second treatment cycle with HBV-GalNAc-siRNA; Figure S10: Differential expression analysis between high and mid titer; Table S1: antibodies used for immunohistochemistry; Table S2: flowcytometry antibody panel.
