# Supplementary material for: Retreatment with HBV siRNA Results in Additional Reduction in HBV Antigenemia and Immune Stimulation in the AAV-HBV Mouse Model
Source: Viruses. 2024 Feb 23;16(3):347. doi: 10.3390/v16030347 (PMC10975807; doi:10.3390/v16030347)
Supplement: Supplementary file 1 [file viruses-16-00347-s001.zip › viruses-2884141-supplementary.pdf]

Retreatment with HBV siRNA Results in Additional Reduction in HBV Antigenemia and Immune Stimulation in the AAV-HBV Mouse Model

Ellen Van Gulck [1,*], Nádia Conceição-Neto [1], Liese Aerts [1], Wim Pierson [1], Lore Verschueren [1], Mara Vleeschouwer [1], Vinod Krishna [2], Isabel Nájera [3] and Frederik Pauwels [1]

[1] Infectious Diseases and Vaccines, Janssen Research and Development, Turnhoutseweg 30, 2340 Beerse, Belgium; nneto@its.jnj.com (N.C.-N.); lieseaerts@hotmail.com (L.A.); wpierso1@its.jnj.com (W.P.); lversch2@its.jnj.com (L.V.); mvleesch@its.jnj.com (M.V.); frpauwel@gmail.com (F.P.)
[2] Infectious Diseases and Vaccines, Janssen Research and Development, 1400 McKean Road, Springhouse
PA 19002, USA; vkrish10@its.jnj.com
[3] Infectious Diseases and Vaccines, Janssen Research and Development, 1600 Sierra Point Parkway, South San Fransisco, CA 94005, USA; isanajera@me.com
* Correspondence: evangulc@its.jnj.com

Supplementary Tables

Supplementary Table S1: antibodies used for immunohistochemistry

Marker	Catalog Number	Supplier	clone
HBcAg	B0586	Dako	polyclonal
HBsAg	Ab859	Abcam	3E7

Supplementary Table S2: Flow cytometry antibody panel.

Marker	Fluorochrome	Catalog Number	Supplier	clone
CD16/CD32	Purified	553142	BD	2.4G2
CD45	BUV-395	564279	BD	30-F11
CD8a	BUV496	750024	BD	53-6.7
CD4	BV-786	563331	BD	GK1.5
CD3	Percpcy5.5	560527	BD	17A2
PD-1	BV-605	563059	BD	J43
LAG-3	BUV737	741820	BD	C9B7W
TIM-3	BB515	567810	BD	5D12
TIGIT	PE	622206	biolegend	A17200C
CD154	PE-Cy7	106512	biolegend	MR1
TOX	A647	568356	BD	NAN448B
FOXp3	PE-CF594	567456	BD	3G3
TCF1	A405	FAB8224V	Rnd systems	#812145

Supplementary figures

Supplementary Figure S1: T-cell gating.

The following gating strategy is followed to determine pre-exhausted, exhausted and terminally exhausted T-cells. Lymphocyte gate was made based on forward scatter (FSC)-side scatter (SSC). Next single cells are selected based on FSC-Area/SSC-Area. Live dead stain was plotted against CD45 to select the living CD45+ cells. Next SSC was plotted against CD3 to select T-cells. CD4 was plotted against CD8 within the CD3 + T cell gate to distinguish between CD8 and CD4 T-cells. In both these gates the expression of PD-1, TOX, TIM-3, LAG-3 and TIGIT was evaluated. Tregs were characterized as FOXp3+ within the CD4 T-cells.

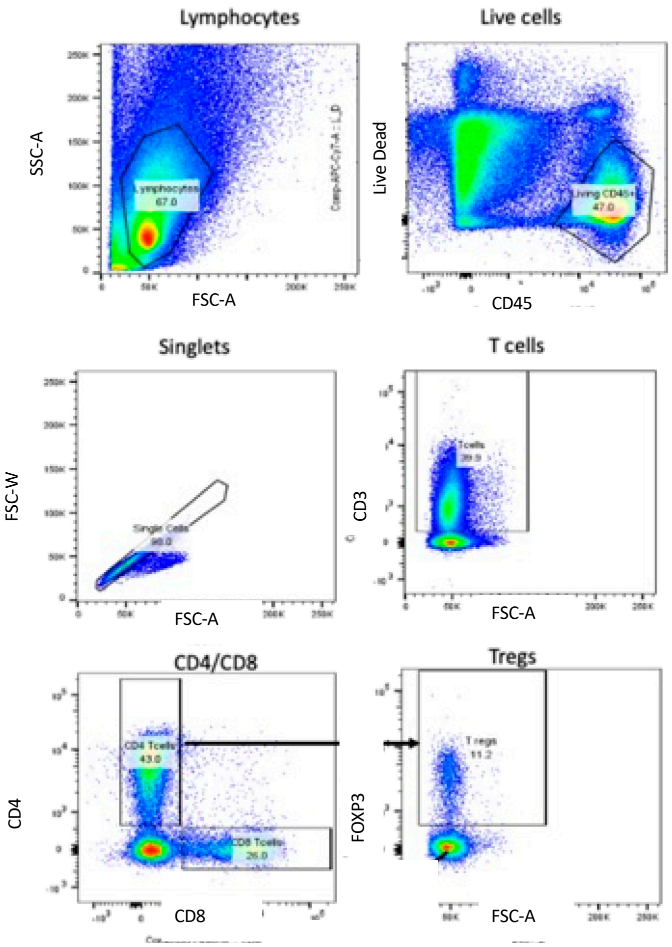

Supplementary Figure S2: HBV DNA in serum of mice transduced with AAV-HBV and treated with GalNac-HBV siRNA

High titer mice, transduced with $3x10^{10}$vge/mice of AAV-HBV (**A**) or Mid titer mice:transduced with $2.5x10^{9}$vge/mice of AAV-HBV (**B**), start treatment twenty-eight days after transduction. The first treatment cycle of 4 doses of 3mg/kg siRNA every 3 weeks is indicated with red dotted lines. The second treatment cycle is indicated with black dotted lines. G1 and G4 received twice a treatment with GalNAc-control-siRNA (grey diamonds). G2 and G5 received first GalNAc-HBV-siRNA and then GalNAc-control-siRNA (grey

triangle). G3 and G6 received twice GalNAc-HBV-siRNA (black squares). Blood was taken on weekly basis to evaluate viral parameters.

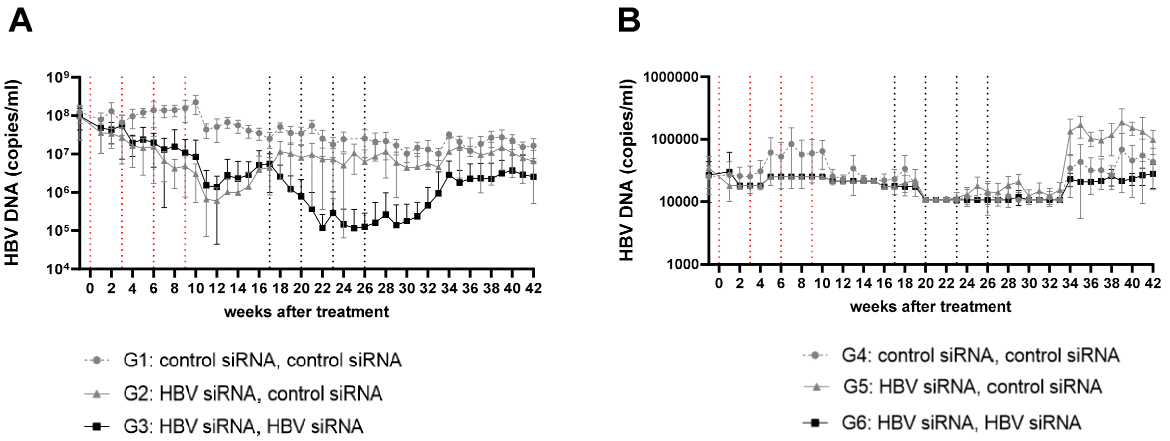

Supplementary Figure S3: Viral parameters in serum and liver of mice transduced with high titer virus and treated twice with control GalNac siRNA

High titer mice are mice transduced with 3x10^{10}vge/mice of AAV-HBV. Twenty-eight days after transduction treatment started. The two-treatment cycle of 4 doses of 3mg/kg siRNA every 3 weeks are indicated with dotted lines. Blood was taken weekly to evaluate viral parameters. (**A**) HBsAg levels, (**B**) HBeAg levels, (**C**) HBs antibodies, (**D**) viral load, (**E**) ALT.

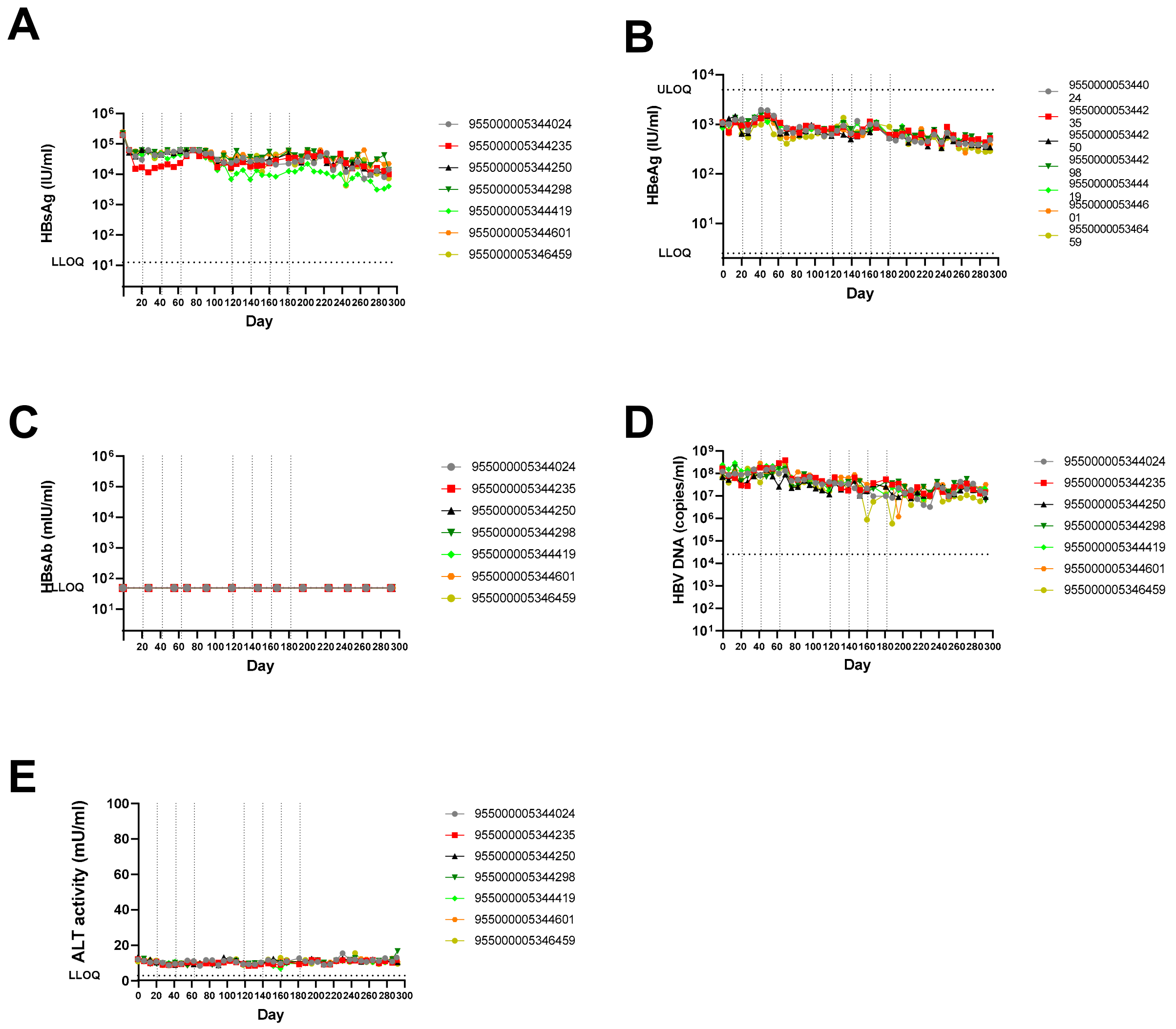

Supplementary Figure S4: Viral parameters in serum and liver of mice transduced with high titer virus and treated with GalNac-HBV-siRNA and with GalNac-control-siRNA

High titer mice are mice transduced with $3x10^{10}$vge/mice of AAV-HBV. Twenty-eight days after transduction treatment started. The treatment cycles of 4 doses of 3mg/kg HBV-siRNA every 3 weeks are indicated with dotted lines. Blood was taken weekly to evaluate viral parameters. (**A**) HBsAg levels, (**B**) HBeAg levels, (**C**) HBs antibodies, (**D**) viral load, (**E**) ALT.

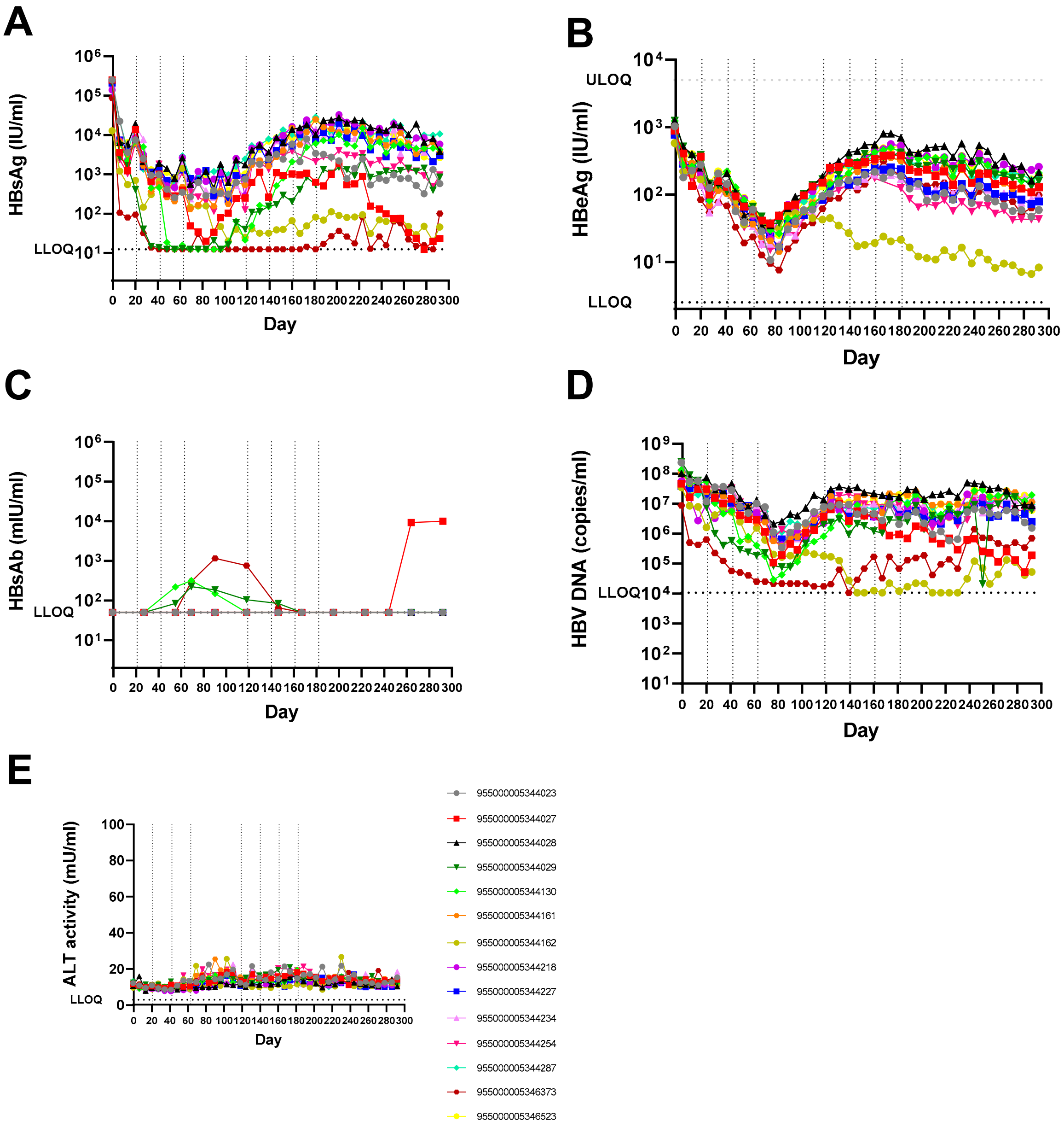

Supplementary Figure S5: Viral parameters in serum and liver of mice transduced with high titer virus and treated twice with GalNac-HBV-siRNA.

High titer mice are mice transduced with $3x10^{10}$vge/mice of AAV-HBV. Twenty-eight days after transduction treatment started. The treatment cycles of 4 doses of 3mg/kg HBV-siRNA every 3 weeks are indicated with dotted lines. Blood was taken weekly to evaluate viral parameters. (**A**) HBsAg levels, (**B**) HBeAg levels, (**C**) HBs antibodies, (**D**) viral load, (**E**) ALT.

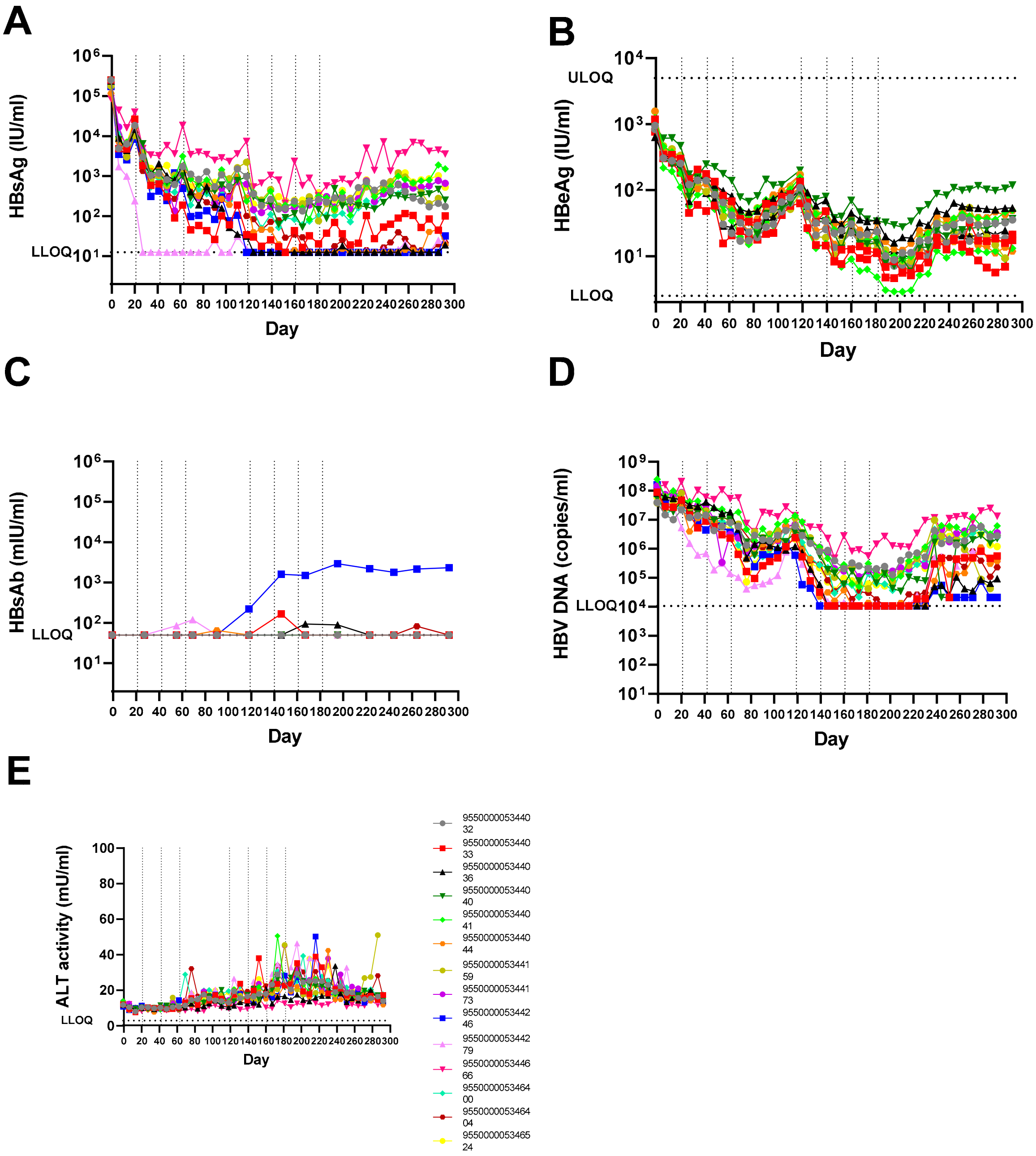

Supplementary Figure S6: Viral parameters in serum and liver of mice transduced with mid titer virus and treated twice with GalNAc-control-siRNA.

Mid titer mice are mice transduced with $2.5x10^9$vge/mice of AAV-HBV. Twenty-eight days after transduction treatment started. The treatment cycles of 4 doses of 3mg/kg HBV-siRNA every 3 weeks are indicated with dotted lines. Blood was taken weekly to evaluate viral parameters. (**A**) HBsAg levels, (**B**) HBeAg levels, (**C**) HBs antibodies, (**D**) viral load, (**E**) ALT.

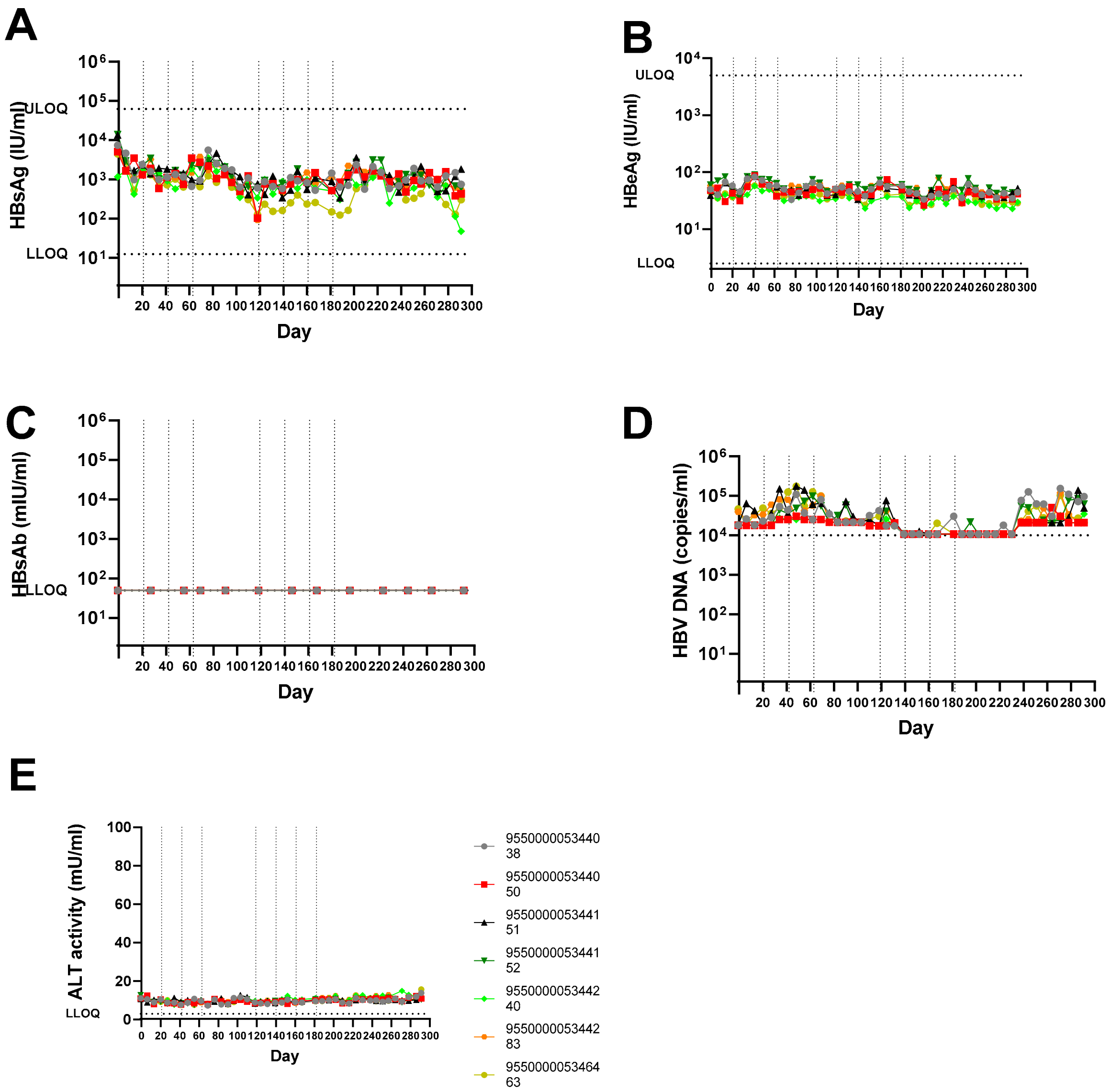

Supplementary Figure S7: Viral parameters in serum and liver of mice transduced with mid titer virus and treated with GalNAc-HBV-siRNA and retreated with GalNAc-control-siRNA

Mid titer mice are mice transduced with $2.5x10^9$vge/mice of AAV-HBV. Twenty-eight days after transduction treatment started. The treatment cycles of 4 doses of 3mg/kg HBV-siRNA every 3 weeks are indicated with dotted lines. Blood was taken weekly to evaluate viral parameters. (**A**) HBsAg levels, (**B**) HBeAg levels, (**C**) HBs antibodies, (**D**) viral load, (**E**) ALT.

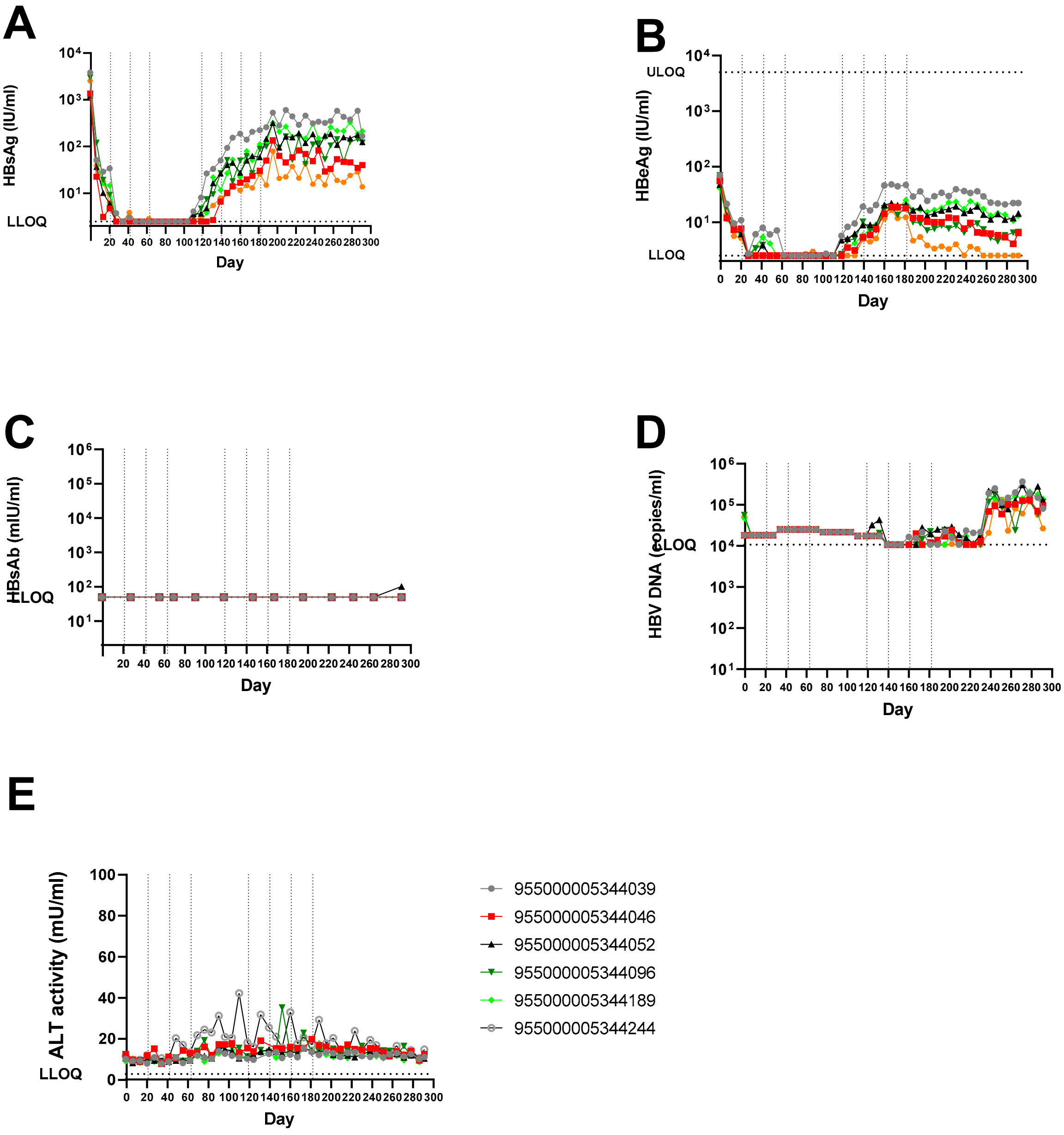

Supplementary Figure S8: Viral parameters in serum and liver of mice transduced with mid titer virus and treated twice with GalNac-HBV-siRNA.

Mid titer mice are mice transduced with 2.5x10^{9}vge/mice of AAV-HBV. Twenty-eight days after transduction treatment started. The treatment cycles of 4 doses of 3mg/kg HBV-siRNA every 3 weeks are indicated with dotted lines. Blood was taken weekly to evaluate viral parameters. (**A**) HBsAg levels, (**B**) HBeAg levels, (**C**) HBs antibodies, (**D**) viral load, (**E**) ALT.

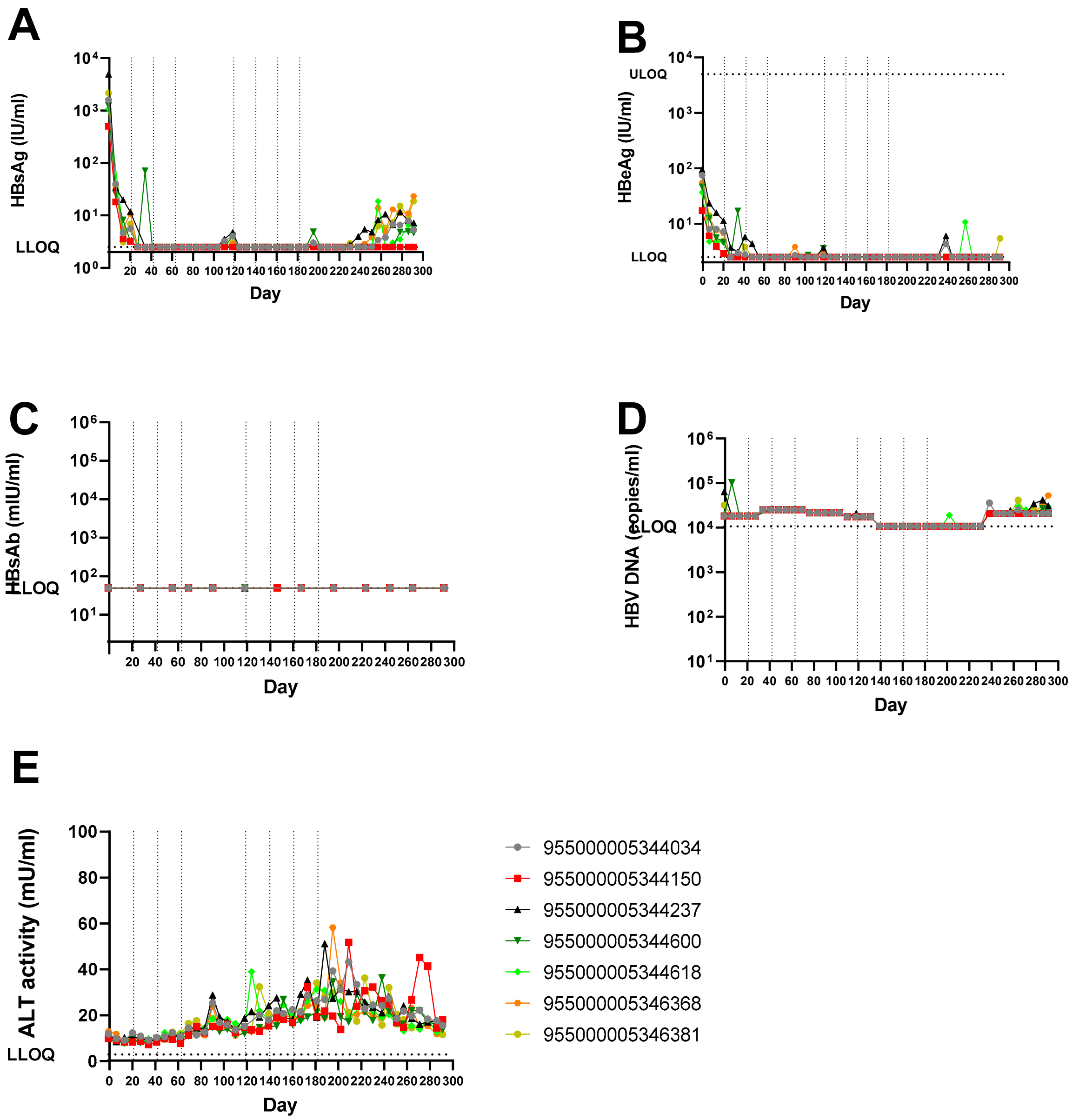

Supplementary Figure S9: Changes from baseline in HBsAg levels during first and second treatment cycle with HBV-GalNAc-siRNA

Changes from baseline were calculated for mice treated with HBV-GalNac-siRNA during the first treatment cycle (A). Slope of decline in HBsAg was calculated using simple linear regression method. The slope of decline was comparable in mid titer (grey triangle, dotted line) and high titer mice (black circle and line). Changes from baseline (defined as last value of HBsAg levels before new cycle of treatment

started) were calculated for mice treated with HBV-GalNac-siRNA during second treatment cycle (B). The slope of decline was significantly different between mid-titer and high titer arm. But this is due to concentrations of HBsAg levels at start of second treatment cycle. Which was just above detection limit in mid titer mice.

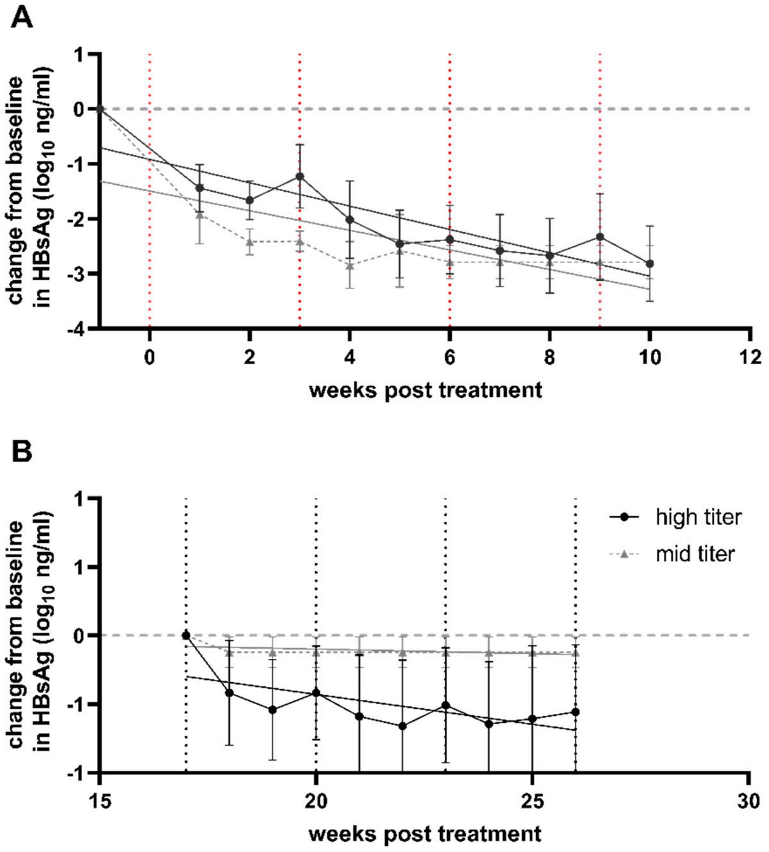

Supplementary Figure S10: Differential expression analysis between high and mid titer

Mid titer mice are mice transduced with 2.5x10^9vge/mice of AAV-HBV. High titer mice are mice transduced with 3x10^{10}vge/mice of AAV-HBV. (**A**) Volcano plot comparing controls from high vs mid titer (**B**) single treated mice with HBV-GalNAc-siRNA from high and mid titer mice and (**C**) retreated mice with two rounds of HBV-GalNAc-siRNA from high and mid titer mice. Colors indicate p-value fdr correction.

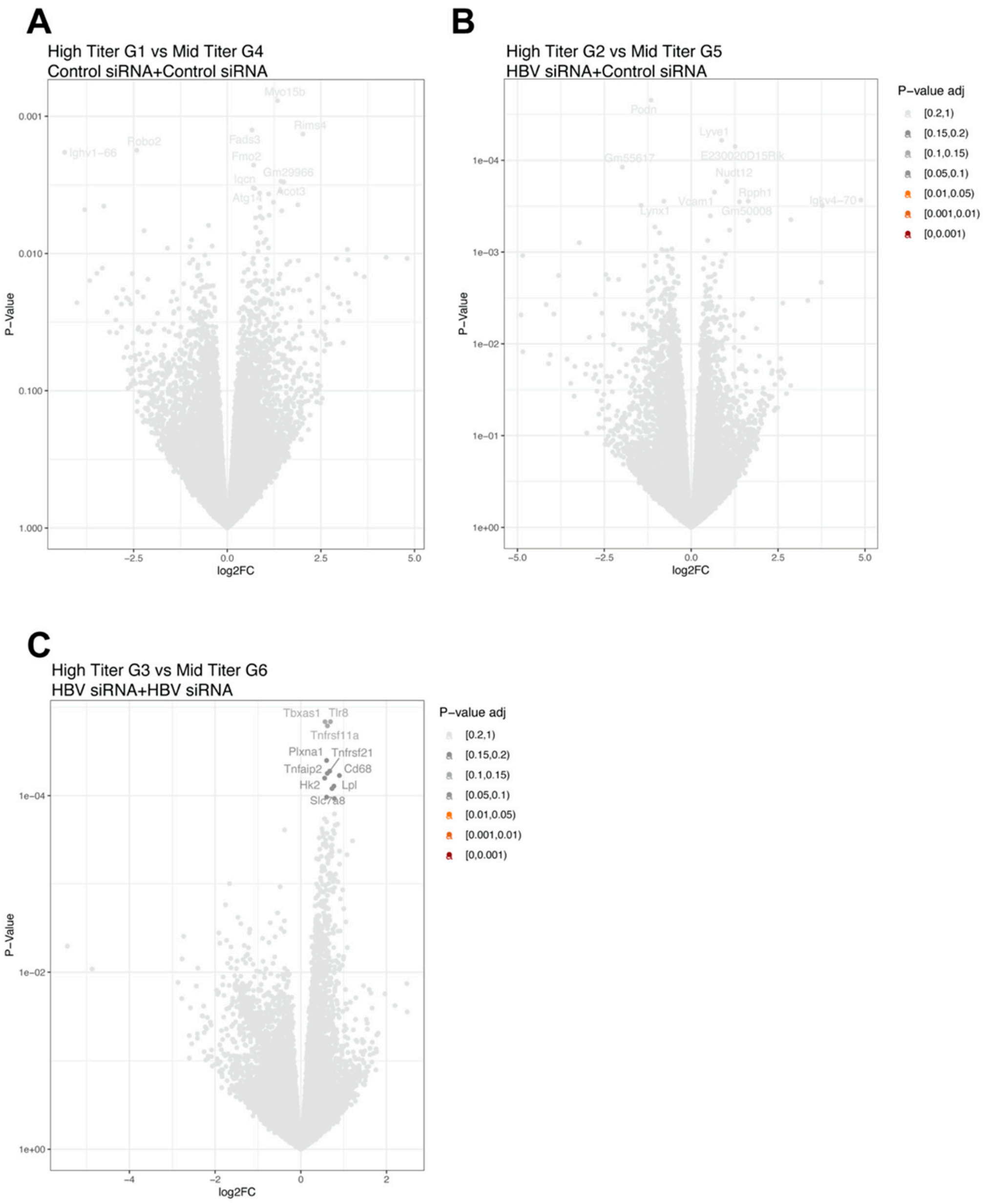
A
High Titer G1 vs Mid Titer G4
Control siRNA+Control siRNA
Myo15b
Rims4
Robo2
Fads3
Ighv1−66
Fmo2
Iqcn
Gm29966
Acot3
Atg14
P−Value
0.001
0.010
0.100
1.000
log2FC
−2.5
0.0
2.5
5.0
B
High Titer G2 vs Mid Titer G5
HBV siRNA+Control siRNA
Podn
Lyve1
Gm55617
E230020D15Rik
Nudt12
Rpph1
Igkv4−70
Vcam1
Lynx1
Gm50008
P−Value
1e−04
1e−03
1e−02
1e−01
1e+00
log2FC
−5.0
−2.5
0.0
2.5
5.0
P−value adj
[0.2,1)
[0.15,0.2)
[0.1,0.15)
[0.05,0.1)
[0.01,0.05)
[0.001,0.01)
[0,0.001)
C
High Titer G3 vs Mid Titer G6
HBV siRNA+HBV siRNA
Tbxas1
Tlr8
Tnfrsf11a
Plxna1
Tnfrsf21
Tnfaip2
Cd68
Hk2
Lpl
Slc7a8
P−Value
1e−04
1e−02
1e+00
log2FC
−4
−2
0
2
P−value adj
[0.2,1)
[0.15,0.2)
[0.1,0.15)
[0.05,0.1)
[0.01,0.05)
[0.001,0.01)
[0,0.001)
